# Impact of rural‐urban residence and deprivation on care pathways for depression disorders among adults in the UK

**DOI:** 10.1111/jrh.70055

**Published:** 2025-07-20

**Authors:** Maxime Inghels, David Nelson, Ros Kane, Mark Gussy, Carl Deaney

**Affiliations:** ^1^ Lincoln Institute for Rural and Coastal Health College of Health and Science University of Lincoln Lincoln UK; ^2^ Marsh Medical Practice Louth UK; ^3^ School of Health and Care Sciences College of Health and Science University of Lincoln Lincoln UK

**Keywords:** access to care, care pathway, depression, deprivation, environmental factors, mental health, rural health

## Abstract

**Purpose:**

To investigate how rurality shapes individual care pathways and health outcomes for depression and to investigate the sociodemographic and economic relationships with urban‐rural variations.

**Methods:**

A retrospective cohort study using routinely collected data from adult patients diagnosed for depression and registered at a general practice in Lincolnshire in the UK. Access and time to access from the onset of depression symptoms to the following care pathway states were described (ie, access to a depression screening tool, confirmed diagnosis, access to treatment and outcomes). Multistate survival analyses were conducted to investigate the effect of the patient's living environment (rural/urban, index of multiple deprivation) on progression through their care pathway for depression.

**Findings:**

Overall, 1,111 patients with depression were included. While access to depression services were lower for patients living in rural areas, they were more likely to experience positive depression outcomes, and more quickly, compared to their urban counterparts. Controlled depression and relapse rates were, respectively, 29% lower and 31% higher among urban residents. The level of deprivation was found to have a limited effect on care access, as well as on depression outcomes.

**Conclusion:**

While accessing care services remains a challenge in rural areas, our study highlights the potential benefits of the rural context in improving depression outcomes and lowering relapse risk. Area‐based deprivation had minimal impact on both care access and depression outcomes. Future mental health programs must tailor their strategies to the unique challenges of urban and rural environments to facilitate more effective interventions.

## INTRODUCTION

Depression is a major and growing public health concern that affects 1 in 6 people in the UK.^1^ The Covid‐19 pandemic and the subsequent cost of living crisis have resulted in unprecedented hazards to mental health globally, with high rates of anxiety, depression, post‐traumatic stress disorder, psychological distress, and stress reported in the general population.^2^ Rural areas, which are home to nearly 10 million UK citizens, are particularly prone to poor health and mental health outcomes.^3^ While rural areas are sometimes viewed as healthy places due to less noise and environmental pollution than found in urban areas, as well as having increased access to nature and green spaces, they are also characterized by an aging population who are more susceptible to loneliness and social isolation which can impact negatively on mental health.^4^, ^5^ Moreover, service accessibility continues to be a central problem in rural areas, and most mental health services are located in towns or cities.^6^, ^7^ Access to mental health services is also challenged by the greater sensibility to stigmatization of mental health and to greater concern about confidentiality among small rural communities. While depression disorders are less prevalent in rural areas, suicide rates are similar between rural and urban areas.^8^, ^9^

In addition to reduced access to mental health services, rural areas are also characterized by a high heterogeneity in terms of sociodemographic population. For instance, around one‐sixth of areas with the worst health and deprivation indicators are rural.^10^, ^11^, ^12^ It has been demonstrated that rurality can exacerbate the effects of socioeconomic disadvantage, poorer service availability, and higher levels of personal risk.^11^ On the other hand, urban areas tend to be more ethnically diverse than rural areas, with ethnic minorities documented as having higher prevalence of mental health conditions and reduced access to care.^13^, ^14^, ^15^ Migration patterns, such as selective migration, also contribute to the differences in well‐being between people living in cities and those in the countryside.^16^

To better understand the challenges of accessing and receiving mental health care, some studies have explored and described care pathways—that is, the journey from the onset of symptoms to accessing treatment and achieving health outcomes.^17^, ^18^ However, many of these studies do not consider the complexity of some care pathways (eg, relapse, treatment interruption) and the potential impact of the living environment. Thus, the purpose of our study is to explore how rurality shapes an individual's care pathway and health outcomes for depression and to investigate the sociodemographic and economic relationships with urban‐rural variations. By investigating the health care pathway for a major mental health condition, this study will contribute to the identification of current gaps in mental health care services, which could support policy makers to address the surge in demand for mental health support.

## METHODS

### Study design and population

We conducted a retrospective cohort study using routinely collected data from patients registered at a general medical practice with 2 facilities in East Lindsey, Lincolnshire, United Kingdom. When a patient moves house, their medical records are typically transferred between their previous and new General Practitioner (GP) practices. This ensures that the patient's complete medical history remains accessible to authorized health care professionals within their current registered practice. All records of adult patients with a depression diagnosis documented between 01/01/2008 and 03/04/2024 were included in our study.

### Data collection

Data collected included individual demographic characteristics, age and sex, as well as characteristics of the living place of the patient: rural‐urban classification and index of multiple deprivation (IMD). The rural‐urban classification and the IMD were obtained from the Office for National Statistics and linked with the patient's postcode.^19^ In case of a missing postcode or gap record, the last known postcode was carried forward. The IMD is the measure of relative deprivation used in England and is a composite measure comprising 7 weighted domains, including income, education, employment, health, crime, housing and services, and living environment.

Clinical data included that collected during medical visits on symptoms linked to depression (eg, anxiety, low mood), medical visits for depression screening or diagnosis, treatment provided (antidepressants, referral to a psychiatrist or other mental health services, psycho or cognitive therapies), social prescribing (eg, referral to physical activity), and depression outcome event (eg, normal mood, depression remission). Symptoms or signs of depression included conditions or situations potentially associated with depression (eg, depressed mood or single episodes of depression, emotional distress, anxiety and negative thoughts, mental health issues, suicide attempts or intentional self‐harm, experiences of abuse, bereavement or grief, and a family history of mental health issues), all of which should prompt an assessment for depression. The list of read code with their label and their categorization (ie, symptoms or signs associated with depression, depression screening, depression diagnosis, psychological therapies, social prescribing, depression outcomes) are presented in the appendices (Appendix S1). The dates when these events were reported were also collected.

### Statistical analysis

Characteristics of the patients were described at their first diagnosis of depression. Access to depression care services (ie, depression screening tool, diagnosis, antidepressants, psycho or cognitive therapy, access to a psychiatrist or mental health nurse, social prescribing), depression outcomes (depression resolved or with controlled symptoms), as well as delay to access those services and outcomes were described.

To understand care pathways and the associated factors, we performed multi‐state Markov models. These investigate factors that are associated with the transition intensity or rate from one given care state to another. The table below describes the different care state transitions considered to describe care pathways for depression (Table 1). Transitions can be upward or backward. The transitions indicated in the table are considered in the models. Direct transitions between 2 states separated by 1 or more intermediate states (eg, a patient moving directly from “having depression related symptoms” state to “diagnosed depression” without any documented “screened” state) are also considered by the model to account for various types of care pathways.

**TABLE 1:** State transition considered for the description of care pathways.

Transition name	Transition state
Screened	From having depression‐related symptoms to being screened for depression
Diagnosed	From being screened for depression to being diagnosed for depression
On antidepressants	From being diagnosed for depression to being on antidepressants
Antidepressants interruption	From being on antidepressants to being diagnosed for depression
Depression controlled	From being on antidepressants to having a depression with controlled symptoms or remission
Depression relapse	From having a controlled depression or remission to being diagnosed for depression

The date of the antidepressant prescription and its expected end date were used to determine when a patient initiated or discontinued antidepressant treatment. Since the model requires a start and end date for each state to identify upward or downward transitions, treatments such as referral to a psychiatrist or other mental health service, psycho or cognitive therapies and social prescribing were not included in the transition states because of the absence of data on when these events ended (ie, for how long patients had used these services).

Following bivariate analyses, multivariable models were conducted to investigate the association between each state transition and place of living (Urban/Rural) adjusted for confounding variables (ie, sex, age, and IMD of the living place). All variables included in the model were time variant, so any individual changes, such as moving from an urban to a rural area, are accounted for in our analyses. To address potential nonconstant transition intensities over time, we examined them across the follow‐up period and stratified by time intervals with constant intensities to meet model assumptions.

All analyses were conducted in R 4.4.1. with the packages *msm* for the model estimators and related confidence intervals.^20^, ^21^

## RESULTS

### Population description, access to services, and timing

Among the 6,772 patients registered on 03/04/2024, 1,515 had been diagnosed for depression, 350 of those episodes happened before 01/01/2008, and 1 depression diagnosis had missing date data. Among the remaining 1,164, 29 were under 18 at depression diagnosis and 24 had a gap or missing value for the address, leading to a final sample of 1,111 individuals.

Every individual included was retrospectively followed for a median time of 13.4 years (Interquartile range—IQR: 8.6‐16.3). During that follow‐up, a total of 2,201 depression events were recorded. Almost all these events were moderate or unspecified depression (2,119/2,201, 96.3%), major or severe and mild depression were rare (43/2,201, 2.0%, and 39/2,201, 1.8%, respectively). Among patients, mobility between urban and rural areas was significant, with 12.6% (140/1,111) of individuals moving from an urban to a rural area and 7.4% (82/1,111) from a rural to an urban area, within 5 years of their initial depression diagnosis. When considering population characteristics at their first depression diagnosis per living area and IMD (Table 2), an imbalance was observed in sex and age, with women more frequently living in urban areas and older people more frequently living in the most deprived rural areas. In 75.4% of cases, the screening tool used was not specified (ie, depression screening using general questions), followed by the Hospital Anxiety and Depression Scale (12.8%) and the EuroQol Anxiety and Depression score (8.5%).

**TABLE 2:** Description of the population study at their first diagnosis for depression (n = 1,111).

	Least deprived areas		Most deprived areas		
	Rural N = 318	Urban N = 145	Rural N = 423	Urban N = 225	Global *P*‐value**^a^**
**Sex**					.03
Female	203 (63.8%)	103 (71.0%)	248 (58.6%)	151 (67.1%)	
Male	115 (36.2%)	42 (29.0%)	175 (41.4%)	74 (32.9%)	
**Age**					.02
40 or less	136 (42.8%)	62 (42.8%)	144 (34.0%)	101 (44.9%)	
Over 40	182 (57.2%)	83 (57.2%)	279 (66.0%)	124 (55.1%)	
**Number of depression symptoms at diagnosis**					0.50
Median [IQR]	1 [0‐3]	1 [0‐2]	1 [0‐2]	1 [0‐3]	
**Depression type**					.60
Depression (moderate or unspecified)	309 (97.2%)	141 (97.2%)	411 (97.2%)	214 (95.1%)	
Mild depression	5 (1.6%)	2 (1.4%)	3 (0.7%)	4 (1.8%)	
Major or severe depression	4 (1.3%)	2 (1.4%)	9 (2.1%)	7 (3.1%)	
**Has benefitted from a screening test**					0.13
Yes	147 (46.2%)	68 (46.9%)	192 (45.4%)	123 (54.7%)	
**Delay between depression screening and first symptoms**	*N = 147*	*N = 68*	*N = 192*	*N = 123*	.60
Median in months [IQR]	67 [6‐142]	57 [9‐124]	48 [2‐132]	67 [6‐127]	
**Has initiated antidepressant**					.40
Yes	282 (88.7%)	126 (86.9%)	382 (90.3%)	207 (92.0%)	
**Delay between antidepressant initiation and diagnosis**	*N = 282*	*N = 126*	*N = 382*	*N = 207*	.60
Median in months [IQR]	0 [0‐7]	0 [0‐5]	0 [0‐16]	0 [0‐13]	
**Psycho or cognitive therapy initiated**				0.20	
Yes	37 (11.6%)	21 (14.5%)	36 (8.5%)	28 (12.4%)	
**Delay between psycho or cognitive therapy initiation and depression diagnosis**	*N = 37*	*N = 21*	*N = 36*	*N = 28*	.20
Median in months [IQR]	0 [0‐30]	10 [0‐49]	22 [0‐52]	9 [0‐33]	
**Seen by a psychiatrist or mental health nurse**					<0.01
Yes	93 (29.2%)	27 (18.6%)	141 (33.3%)	82 (36.4%)	
**Delay between being seen by a psychiatrist or mental health nurse and diagnosis**	*N = 93*	*N = 27*	*N = 141*	*N = 82*	.02
Median in months [IQR]	0 [0‐33]	1 [0‐47]	0 [0‐4]	0 [0‐8]	
**Benefited from social prescribing**				0.30	
Yes	120 (37.7%)	58 (40.0%)	170 (40.2%)	104 (46.2%)	
**Delay between social prescribing and diagnosis**	*N = 120*	*N = 58*	*N = 170*	*N = 104*	.001
Median in months [IQR]	29 [0‐76]	41 [17‐81]	13 [0‐60]	18 [0‐61]	
**Had their depression controlled or in remission**					.01
Yes	175 (55.0%)	62 (42.8%)	246 (58.2%)	126 (56.0%)	
**Delay between controlled depression and diagnosis**	*N = 175*	*N = 62*	*N = 246*	*N = 126*	<.001
Median in months [IQR]	58 [18‐116]	96 [35‐137]	56 [7‐97]	80 [41‐119]	

*Note*: Italic values signifies specifying change in the sample size.

^a^ Pearson's Chi‐squared test; Kruskal‐Wallis rank sum test; Fisher's exact test. The global *P*‐value tests for differences in each variable across the 4 groups.

While overall access to antidepressants was widespread (997/1,111, 89.7%), access to screening (530/1,111, 47.7%), psychiatrist or mental health nurse (343/1,111, 30.9%), psycho or cognitive therapies (122/1,111, 11.0%), and social prescribing (452/1,111, 40.7%) remained low.

When comparing patients living in urban areas at their first depression diagnosis with those diagnosed in rural areas, access to screening tests (191/370, 51.6% vs 339/741, 45.7%, *P* = .06), psycho or cognitive therapy initiation (49/370, 13.2% vs 73/741, 9.9%, *P* = .09), and social prescribing (162/370, 43.8% vs 290/741, 39.1%, *P* = .14) were higher in urban areas, close to the statistical significant threshold (ie, 0.05). When accessing those services, the time to access were similar between urban and rural areas.

When stratifying by IMD, access to screening tests, psychotherapy or cognitive therapy, social prescribing, or antidepressants were similar between the most and least deprived areas (Table 2). Delays in accessing care services were also similar, except for access to a psychiatrist, or mental health nurse (median: 0 [0‐6] vs 0 [0‐35] months, *P* < .01) and social prescribing (13 [0‐61] vs 36 [0‐77] months, *P* < .001), both of which were shorter for populations diagnosed in the most deprived areas compared to those in the least deprived areas.

Regarding the outcome of the first depression diagnosis, controlled depression or remission was more frequent in rural areas compared to urban areas (421/741, 56.8% vs 188/370, 50.8%, *P* = .07) and was achieved more quickly (57 [10‐104] vs 86 [39‐124] months, *P* < .001).

Controlled remission was also more frequent among patients diagnosed in high‐deprivation areas (372/648, 57.4% vs 237/463, 52.1%, *P* = .05). However, the delay between depression diagnosis and these positive outcomes was similar between the most and least deprived areas (62 [17‐105] vs 68 [25‐121] months, *P* = .14).

### Care pathways analyses

**FIGURE 1:**
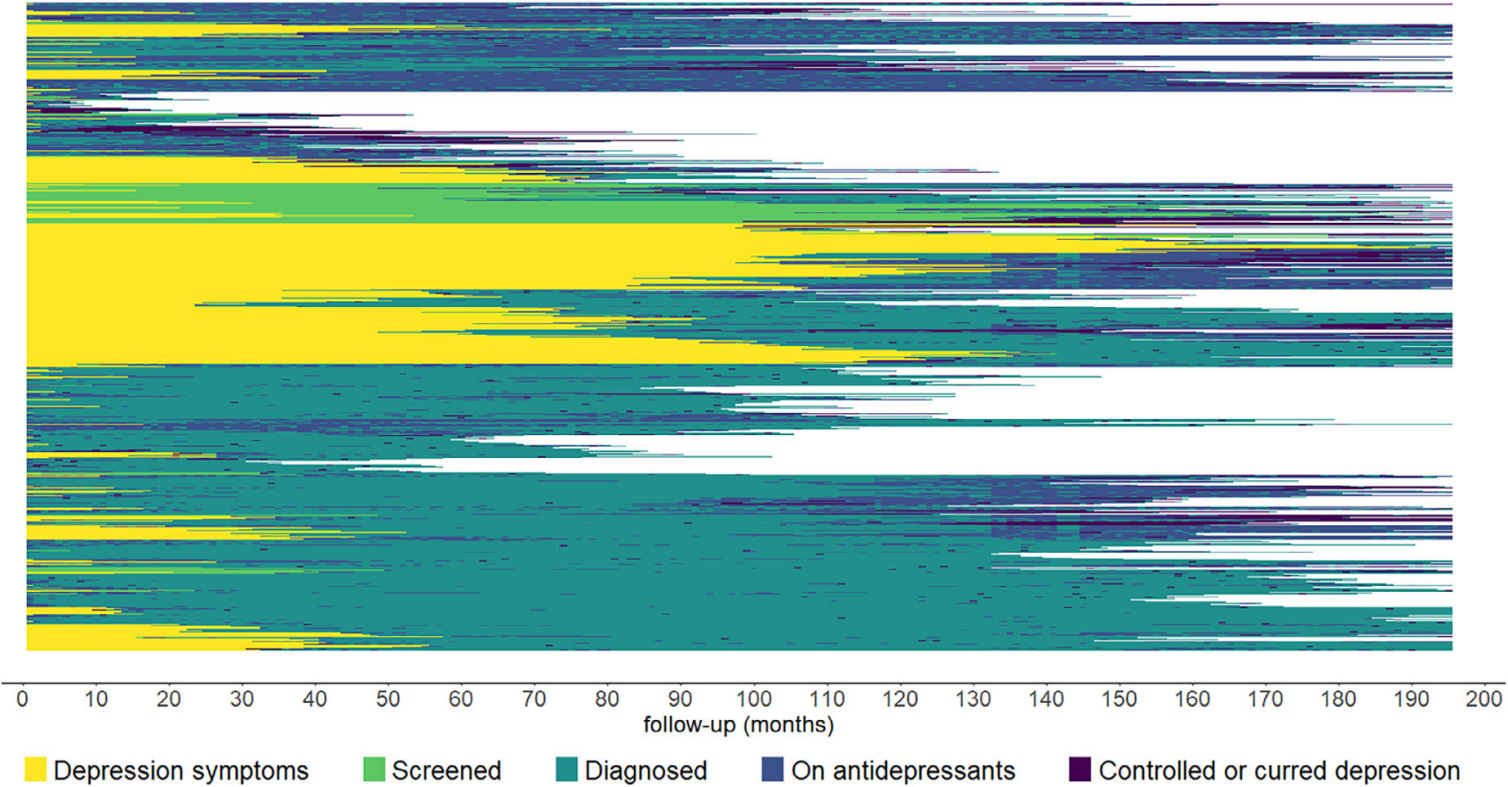
Sequential description of care state transition for each patient (n= 1,111).

Previous analysis has described care services access and outcome and delay in accessing those services. However, the previous analysis does not take into account the fact that some individuals have changed their living areas and the dynamic aspect of the care pathway progressions (eg, stopping antidepressant treatment, relapse) and the time spent in each state. To illustrate the complexity of care pathways, we drew the pathway sequence (Figure 1). Each line represents the care pathway of one given patient, with the initial state of their pathway starting from the left side of the chart. We can observe a vast number of care pathways, with some patients being rapidly diagnosed and having a controlled depression (short lines with dark blue at the end on the chart), some who are diagnosed at a late stage (represented by a long yellow line before turning to dark green), or some interrupting their antidepressants treatment (transition from blue to dark green).

To account for the different state transitions and time variable of the co‐variables of interest (ie, sex, age, living area, IMD), multistate Markov models were conducted to understand the predictors of these transitions (Table 3).

**TABLE 3:** Multistate Markov models investigating states’ transitions for depression care pathways.

State transition	HR	aHR
**Sex**, Men (*vs Women)*		
Screened	0.95 [0.79‐1.13]	0.94 [0.79‐1.12]
Diagnosed	0.62 [0.52‐0.72]	0.67 [0.56‐0.78]
On antidepressants	0.76 [0.72‐0.81]	0.76 [0.72‐0.80]
Antidepressants interruption	0.89 [0.83‐0.94]	0.92 [0.86‐0.98]
Depression controlled	1.61 [1.41‐1.84]	1.56 [1.37‐1.78]
Depression relapse	0.91 [0.80‐1.03]	0.92 [0.80‐1.05]
**Age**, Over 40 *(vs 40 or less)*		
Screened	1.22 [1.03‐1.45]	1.18 [1.00‐1.40]
Diagnosed	0.48 [0.41‐0.56]	0.44 [0.38‐0.53]
On antidepressants	1.09 [1.03‐1.16]	1.12 [1.05‐1.18]
Antidepressants interruption	0.71 [0.67‐0.75]	0.72 [0.68‐0.77]
Depression controlled	0.95 [0.81‐1.11]	0.94 [0.80‐1.10]
Depression relapse	0.65 [0.55‐0.76]	0.66 [0.57‐0.78]
**Living area**, Urban *(vs Rural)*		
Screened	0.84 [0.71‐1.00]	0.83 [0.70‐0.99]
Diagnosed	0.87 [0.74‐1.03]	0.71 [0.60‐0.85]
On antidepressants	1.09 [1.03‐1.15]	1.08 [1.02‐1.14]
Antidepressants interruption	1.48 [1.40‐1.57]	1.48 [1.40‐1.57]
Depression controlled	0.70 [0.60‐0.83]	0.71 [0.61‐0.84]
Depression relapse	1.41 [1.20‐1.66]	1.31 [1.11‐1.54]
**IMD**, Most deprived *(vs least deprived)*		
Screened	1.17 [0.99‐1.39]	1.17 [0.99‐1.39]
Diagnosed	0.91 [0.77‐1.06]	0.98 [0.83‐1.15]
On antidepressants	0.93 [0.88‐0.98]	0.92 [0.87‐0.97]
Antidepressants interruption	0.82 [0.77‐0.87]	0.81 [0.77‐0.86]
Depression controlled	1.16 [1.01‐1.33]	1.15 [1.00‐1.32]
Depression relapse	1.06 [0.92‐1.21]	1.08 [0.94‐1.24]

*Note*: Because transition intensities from one state to another were not constant over time, we considered different transition intensities for the first 12 months, 12‐130, and over 130 months of follow‐up. Model results were adjusted accordingly.

Abbreviations: aHR, adjusted hazard ratio; HR, hazard ratio.

After adjusting for all other co‐variables, screened men were less likely to be diagnosed (adjusted HR 0.67 [0.56‐0.78]), and those diagnosed were less likely to be on antidepressants (aHR 0.76 [0.72‐0.80]). However, treated men were more likely to have their depression controlled (aHR 1.56 [1.37‐1.78]). Being over 40 years old (vs 40 or less) meant a greater likelihood of being on antidepressants when diagnosed (aHR 1.12 [1.05‐1.18]) and less likelihood of experiencing a depression relapse after having their depression controlled (aHR 0.66 [0.57‐0.78]).

Patients living in urban areas were less likely to be diagnosed after screening (aHR 0.71 [0.60‐0.85]). Rates of controlled depression and relapse were, respectively, 29% lower (aHR 0.71 [0.61‐0.84]) and 31% higher (aHR 1.31 [1.11‐1.54]) in urban populations compared to rural ones. Patients living in urban areas were also more likely to initiate antidepressant after diagnosis (aHR 1.08 [1.02‐1.14]), but they were more likely to interrupt this treatment (aHR 1.48 [1.40‐1.57]).

No significant effect of deprivation was found for screening, diagnosis, controlled depression, or relapse in the multivariate model. Rates of antidepressant initiation and interruption were lower in the most deprived areas (aHR 0.92 [0.87‐0.97] and aHR 0.81 [0.77‐0.86], respectively).

## DISCUSSION

Our results showed that a patient's place of residence, whether rural or urban, impacts their care pathway and depression outcomes. While access to depression care was lowest for people diagnosed in rural areas, patients living in these areas were more likely to experience positive depression outcomes, and more quickly, compared to those in urban areas. The level of deprivation was found to have a limited effect on care access as well as on depression outcomes.

One of our key findings was that people from rural areas experienced better depression outcomes. Controlled depression rates were found to be 29% lower among urban residents, while depression relapse rates were 31% higher among them. This result confirms previous UK studies that have found better mental health among rural, compared to nonrural residents.^8^, ^22^ While urban areas have an overall better access to mental health services,^6^, ^7^ they can be a more stressful environment than rural areas due to increased stimulus levels (eg, density, crowding, noise, disarray, pollution).^23^, ^24^ Urban residents can also have diminished access to nature, fewer opportunities to exercise, and reduced leisure time due to higher time spent at work and commuting, which can negatively impact their mental health. Rural residents tend to have stronger community ties and be more involved in community activities.^25^, ^26^

Another significant result of our analysis was the limited influence of area‐level deprivation on an individual's care pathway for depression when adjusted for other covariates. While depression is highly prevalent in deprived areas, the influence of area‐based deprivation on mental health care access and outcomes is mixed in the recent literature.^27^, ^28^, ^29^, ^30^, ^31^ A recent study of 144 psychological services in England found a higher prevalence of common mental disorders in deprived areas, but also that these areas tend to have higher demand and supply of psychological care.^31^ Notably, this study reported that low access to psychological treatment in an area was primarily predicted by a low prevalence of common mental disorders. These findings may explain our observation of higher delays in accessing some depression care services and lower recovery rates among patients diagnosed with depression in an affluent area in our unadjusted analysis; affluent areas, having lower mental disorder prevalence, may consequently have lower mental health service coverage.

With the exception of antidepressants, access to other depression treatments were found to be particularly low. Psycho or cognitive therapies were the lowest utilized service, used by only 11% of patients, even though this is the first intervention choice for depression recommended by the National Institute of Health and Care Excellence—NICE.^32^ GPs’ preference for antidepressant prescriptions is described as being due to time constraints, lack of accessible alternative management options, cost of prescribing, and misconception about mental health and non‐drug‐based therapies.^33^ Psycho or cognitive therapy attendance frequency in our study might also be underestimated as access to these services can be initiated by the patient without GP referral. In addition, screening tools were used in fewer than half of depression diagnoses, with general questions being the most common method of screening. Given the lower sensitivity of shorter or more general questioning for depression, this suggests that a significant proportion of patients with depression may not be adequately screened or properly diagnosed.^34^

Our study has several limitations. Although some originated from various parts of the UK, most patients were from the county of Lincolnshire, which limits the representativeness of our results. Some care services, such as talking therapies or social activities, could have been accessed without a GP referral and are thus potentially underestimated in our results. Because of their absence of address records, homeless people were not included in our analyses. As only registered patients were available, deceased patients’ data could not be accessed, and thus, suicide could not have been considered in our analysis. Due to the lack of data on the duration of psychological therapies, prescribed social activities, or consultations with other mental health professionals, it was not possible to include these types of treatments in our model.

Overall, our study is one of the first to fully consider the care pathway for depression and the interlink of rural‐urban residence and deprivation for a large retrospective cohort in the UK. Our results confirm the important impact of living environment on mental health care access and outcomes, particularly the rural‐urban context, which emerged as a stronger factor than area‐level deprivation.

## CONCLUSION

While accessing care services remains a challenge in rural areas, our study highlights the potential benefits of the rural context in improving depression outcomes and lowering relapse risk. Area‐based deprivation had minimal impact on both care access and depression outcomes. Future mental health programs must tailor their strategies to the unique challenges of urban and rural environments to facilitate more effective interventions.

## FUNDING INFORMATION

This study was funded by the Sir Halley Stewart Trust (Grant reference: 3061) (https://www.sirhalleystewart.org.uk/). The funders had no role in study design, data collection, data analysis, data interpretation, or writing of the article.

## CONFLICT OF INTEREST STATEMENT

All authors declare: no support from any organization for the submitted work; no financial relationships with any organization that might have an interest in the submitted work in the previous 3 years, no other relationships or activities that could appear to have influenced the submitted work. In addition, none of the authors have current or previous professional affiliation with the funder.

## TRANSPARENCY DECLARATION

The corresponding author confirms having access to all the data in the study and had final responsibility for the decision to submit for publication. The corresponding author affirms that the manuscript is an honest, accurate, and transparent account of the study being reported; that no important aspects of the study have been omitted; and that any discrepancies from the study as planned (and, if relevant, registered) have been explained.

## ETHICAL CONSIDERATIONS

This study was an audit and service assessment of previously collected data and did not require NHS Research Ethics Committee review. The review examined data from the electronic record system, originally gathered for routine clinical work. The data were used solely for this retrospective review. All data utilized were deidentified and anonymized before analysis to protect participant confidentiality and stored securely in compliance with the UK Data Protection legislation. Access to the data was restricted to authorized personnel who already had access as part of their role. All findings are reported accurately and honestly, without fabrication, falsification, or inappropriate data manipulation.

## Supporting information



Supporting Information
