# Supplementary material for: Impact of rural‐urban residence and deprivation on care pathways for depression disorders among adults in the UK
Source: J Rural Health. 2025 Jul 20;41(3):e70055. doi: 10.1111/jrh.70055 (PMC12277484; doi:10.1111/jrh.70055)
Supplement: Supplementary file 1 — Supporting Information [file JRH-41-0-s001.docx]

Appendix 1. Clinical and non-clinical event classification of each care state

| **Care step** | **Event** | **Label and read code** |
| --- | --- | --- |
| **Symptoms or sign associated with depression** | |  |
|  | Depressed mood or single episodes of depression | Depressed mood (XE0re); Feels unwell (16E..); (Depressed (& symptom)) or (unhappy) (XE0uv); Symptoms of depression (XaImU); C/O - feeling depressed (XM0CR); History of postnatal depression (XaNg1); (Depressed) or (C/O feeling: [depressed] or [unhappy]) (1B17.); Feeling lonely (XE0rk); C/O - feeling unhappy (XM0CS); Brief depressive reaction NOS (E290z) |
|  | Emotional state , anxiety and negative thoughts | Stress at home (XE0pM); Anxiety attack (Xa7kB); Anxiety state NOS (E200z); Suicidal thoughts (1BD1.); Stress-related problem (1B1L.); Anxiety disorder (E200.); Emotional state observations (Ua16B); Feeling anxious (XE0rb); Level of anxiety (Xa3Xk); Acute stress reaction (XE1Ym); [X]Anxiety disorder, unspecified (Eu41z); Anxiety state unspecified (E2000); Emotional problems (Ua18K); At risk of DSH - deliberate self harm (XaIuV); Moderate suicide risk (XaIeW); Stress and adjustment reaction (XM0As); Generalised anxiety disorder (E2002); Chronic anxiety (E2004); Mood swings (Xa3Xg); Worried (1BK..); Post-traumatic stress disorder (X00Sf); [D]Malaise and fatigue NOS (R007z); Mood observations (X760o); C/O tearfulness (XaCIO); Feeling frustrated (Ua164); [D]Malaise (R0070); Irritability and anger (XM1Ae); Suicide risk (Ua1WE); Mood disorder (XE1Xy); Emotionally unstable personality disorder (E2152); High suicide risk (XaIeV); Thoughts of deliberate self harm (XaIux); Loss of hope for the future (X761G); Planning suicide (X75yS); Acute stress reaction NOS (XE1Yn); Malaise/lethargy (XE0ql); Restlessness and agitation (XM1Af); [X]Other anxiety disorders (Eu41.); Loss of capacity for enjoyment (X761E); Feeling irritable (XE0rc); Cyclic mood swings (XaAyL); Emotional upset (XaC2u); Variability of mood (Xa1eL); Recurrent anxiety (E2005); [X]Mixed anxiety and depress disord (& mild anxiet depressn) (Eu412); Moody (Xa3Xf); Malaise and fatigue (XM06l); Anxiousness (& symptom) (1B13.); [D]Malaise and fatigue (R007.); Difficulty controlling anger (Xa3HT); Difficulty controlling emotions (Xa3HW); Feeling angry (XM016); Suicidal (XE0rg); Feeling of hopelessness (Xa3Xh); Separation anxiety disorder (E2920); Confusional state (Xa3f0); Anxiety about dying (Xa0XQ); Unstable mood (X7617); [X]Other mixed anxiety disorders (Eu413); Elevated mood (X760w); Volatile Mood (Ua1r7); Parental anxiety (Xa7k9); [V]Other physical and mental strain related to work (ZV4By); Emotional immaturity (X765b); C/O - feeling unhappy (XM0CS); Threatening suicide (Xa3FF); Other post-traumatic stress disorder (E29y1); [X]Other specified anxiety disorders (XE1Zj); Nervousness (XE0ra); Anxiety about body function or health (X761T); Emotional behavioural difficulties (Xac0U); Emotionally apathetic (Ua1r0); [D]Irritability and anger (R00z8); Unable to control emotions (Xa3HV); Feeling agitated (Ua15v); [D]Nervousness (R2y2.); Chronic post-traumatic stress disorder (XaEFB); Separation anxiety (XM00G); Acute confusional state NOS (E030z); Blunting of mood (Xa00z); Guilty ideas (XE0s7); Irritable (& symptom) (1B15.); [X]Elective mutism (Eu940); Feeling grief (Ua15r); [X]Behavioural/emotional disords onset childhood/adolescence (Eu9..); Labile mood (Xa3Xd); Anxiety about altered body image (Ua1qp); Other acute stress reactions (E283.); Organic anxiety disorder (X00RP); Feeling suicidal (Ua1XF); Stress reaction causing mixed disturbance of emotion/conduct (E284.); Apathy (XM013); Feeling upset (Ua168); Feeling of loss of feeling (Xa02E); Listlessness (X760S); [X]Tic disorders (Eu95.); Acute post-trauma stress state (E2831); [X]Emotional disorders with onset specific to childhood (Eu93.); Feeling isolated (XaAxa); Difficulty understanding own emotions (Xa3WM); Behavioural, emotional and social difficulties (Xac0T); Harmful thoughts (1BD..); [X](Conduct dis:[variants])(gang offen)(grp stealing)(truan) (Eu912); Feeling tense (XC0CP); Carer stress syndrome (Xa7mz); Mental distress (XM012); Suicidal (& [symptom]) (1B19.); Dysphoric mood (XaKUk); [X]Reaction to severe stress, unspecified (Eu43z); Becomes angry very easily (Ua18O); Reactive confusion (E132.); Other acute stress reaction NOS (E283z); Unable to control anger (Ua18N) |
|  | Other mental health issues | Gender identity disorder (X00TG); C/O - panic attack (XaIo7); Panic attack (Ua1qS); [X]Agoraphobia (& [no hist panic disord][with panic disord]) (Eu400); Fear of transport (X761t); Anorexia nervosa (E271.); Adjustment disorder (E29..); Vascular dementia (XE1Xs); Dementia (X002w); Stress and adjustment reaction (XM0As); Panic disorder (XE1Y7); Obsessive-compulsive disorder (E203.); [X](Lack/loss sex desire)(frig)(hypoact sex dis)(lack libid) (Eu520); Personality disorder NOS (XE1YM); Paranoid schizophrenia (E103.); Asperger syndrome (X00TP); Dissociative reaction unspecified (E201A); [X]Bipolar affective disorder, unspecified (Eu31z); Bipolar disorder (X00SM); Dysthymia (E2112); Autistic spectrum disorder (X00TM); Mild learning disability (XaREt); Attention deficit hyperactivity disorder (XE2Q6); Claustrophobia (E2028); Delirium (XE1Xv); Needle phobia (X00SY); Psychotic disorder (X00S6); Emotionally unstable personality disorder (E2152); Seasonal affective disorder (X761L); Reading disorder unspecified (E2F00); Personality disorder (XE2b6); Mental health disorder (E....); Fear of flying (X762G); Neurasthenia (XE1YD); [X]Dementia in Parkinson's disease (Eu023); Eating disorder (X00Sx); Mental disorders NOS (Ez...); Child attention deficit disorder (E2E0.); Social phobia (X00SW); Agoraphobia (X00SV); Alzheimer's disease (F110.); [X]Dementia in Alzheimer's disease (Eu00.); Fear of falling (XaIvf); [X]Unspecified nonorganic psychosis (XE1ZU); Paranoid personality disorder (XE1YF); Dissocial personality disorder (XE1YJ); FH: Schizophrenia (1284.); Fear of pregnancy (E202E); Delusional disorder (XE1ZO); FH: Senile dementia (1281.); Recurrent major depressive episodes, moderate (E1132); Bulimia nervosa (XE1Yk); Schizoaffective disorder (Eu25.); Mixed disorder of psychological development (E2F5.); Agoraphobia with panic attacks (E2021); Compulsive eating dis (& [bulimia incl non-organic overeat]) (E2751); Flying phobia (X762H); School phobia (XM0Ak); Mixed dementia (Y23fb); Paranoid psychosis NOS (E12z.); Adjustment reaction NOS (E29z.); [X]Mental and behavioural disorders due to use of alcohol (Eu10.); Schizophrenia (Eu20.); Non-organic psychosis NOS (XE1Y5); Drug-induced psychosis (E02..); Fear of fainting (X761c); [X]Vascular dementia, unspecified (Eu01z); Subcortical vascular dementia (X003T); Anankastic personality disorder (XE1YH); Dental phobia (E202C); Psychogenic dyspepsia (E2644); Nondelusional dysmorphophobia (Xa0lG); Specific fear (X7627); Neurotic disorder NOS (XE1YE); [X]Eating disorders (Eu50.); Acute situational disturbance (E2830); [X]Obsessive-compulsive disorder, unspecified (Eu42z); Obsessional thoughts (X762c); Schizoaffective disorder, mixed type (XE2b8); Unspecified bipolar affective disorder (E117.); FH: Alzheimer's disease (XaIkh); Fear of insects (Xa3Vk); Phobic disorder NOS (XE1YB); Gilles de la Tourette syndrome (E2723); Adjustment reaction with anxious mood (E2924); (Behaviour disorder) or (disturbance of conduct NEC) (E2C..); Single manic episode, mild (E1101); Panic (Xa3Vj); Multi-infarct dementia (Xa0lH); Schizophrenia NOS (E10z.); [X]Dissociative [conversion] disorders (Eu44.); Moderate learning disability (XaQZ3); Adjustment reaction with mixed disturbance of emotion (E292y); [X]Dementia in Alzheimer's dis, atypical or mixed type (Eu002); Bipolar affective disorder , current episode mixed (Eu316); Other paranoid states (E12y.); Dementia of frontal lobe type (Xa0sE); [X]Eating disorder, unspecified (Eu50z); Masochistic personality disorder (E21y6); Paranoid schizophrenia NOS (E103z); Premenstrual dysphoric disorder (XSGp0); [X]Schizoaffective disorder, unspecified (Eu25z); Dissociative convulsions (E2015); [X]Developmental disorder of scholastic skills, unspecified (Eu81z); Dementia in Alzheimer's disease with late onset (X0030); Persistent delusional disorder (X00SA); Elective mutism (XE2uz); Lack or loss of sexual desire (XE2bA); Specific phobia (X00SX); Bipolar affective disorder, currently manic, mild (E1141); Mixed disorder of scholastic skills (Eu813); Developmental disorder of scholastic skill (X00TL); Schizophrenic disorders (E10..); Bipolar affect disord, now depressed, severe with psychosis (E1154); Borderline personality disorder (E21y2); Fear of dentist (Ua1qa); Chronic schizophrenic (E1002); Bipolar affective disorder, current episode manic (E114.); Trichotillomania (Eu633); Other mental disorders (XaB97); Severe learning disability (XaQZ4); Other paranoid states NOS (E12yz); Cyclothymia (E2113); Bipolar affective disorder, current episode depression (E115.); Mixed bipolar affective disorder (E116.); Dermatitis artefacta (M184.); Habit and impulse disorder (Eu63.); Somatization disorder (E20y0); Other conversion disorder (XE1Y8); Charles Bonnet syndrome (F49z0); Acute transient psychotic disorder (X00SC); Psychotic episode NOS (X00Qx); Fear of crowds (E2029); Situational panic attack (X00SZ); Fear of needles (Xa1Ev); Personality disorders (& neurotic) (E21..); Obsessive-compulsive disorder NOS (E203z); [X]Gender identity disorders (Eu64.); Other affective psychosis NOS (E11zz); Disorder of psychological development (Eu8..); Mania (X00SJ); [X]Mental and behavioural disorders due to use cannabinoids (Eu12.); Pathological gambling (E2C31); Bipolar II disorder (X00SN); Depersonalisation syndrome (E206.); Brief depressive adjustment reaction (E290.); Other phobias (XaB96); Hypochondriacal disorder (E207.); Psychosis: [nonorganic NOS] or [episode NOS] (E13z.); Bipolar I disorder (XaY1Y); [X]Mixed and other personality disorders (Eu61.); [X]Mania with psychotic symptoms (XE1ZV); Bipolar affective disorder, currently depressed, NOS (E115z); Paranoid disorder (E12..); Compulsive gambling (Xa2ke); (Panic disorder) or (panic attack) (E2001); Avoidant restrictive food intake disorder (Y305e); [X]Gender identity disorder, unspecified (Eu64z); Gender dysphoria (XSEDT); Psychogenic vomiting NOS (E2754); Rebound mood swings (E11z1); Neurotic disorder (E20..); Tic disorder (Xa9BB); Atypical anorexia nervosa (X00Sz); Explosive personality disorder (X00T6); Hypomania (X00SL); Self-induced purging (X76dF); Schizoaffective disorder, depressive type (XE2un); [X]Personality disorder, unspecified (Eu60z); Unspecified disturbance of conduct (E2Cz.); Unspecified bipolar affective disorder, NOS (E117z); Dependent personality disorder (XE1YI); Schizoaffective schizophrenia NOS (E107z); Acute exacerbation of chronic paranoid schizophrenia (E1034); Schizoaffective schizophrenia (E107.); [X] (Bulimia nervosa) or (bulim NOS) or (hyperorexia nervos) (Eu502); Fear of enclosed spaces (X762C); Hyperkinetic syndrome NOS (E2Ez.); Obsessional neurosis (E2031); Obsessional thoughts of causing harm to others (XM001); Other and unspecified affective psychoses (E11z.); Unspecified puerperal psychosis (E03y3) |
|  | Suicide attempt and intentional self-harm | Suicide and selfinflicted injury by cutting and stabbing NOS (TK6z.); [X](Intentional self-harm) or (suicide) (U2...); Self-harm (X766J); Attempted suicide - jumping from a high place (Xa3jH); Suicide attempt (Ua18F); Attempted suicide - cut/stab (Xa3jG); [X]Intentional self harm by other specified means (U2y..); [X]Intentional self harm by sharp object (U29..); Attempted suicide NOS (Xa3jI); Suicide and selfinflicted injury (XE22H); Cutting self (X766V); [X]Intentional self harm by unspecified means (U2z..); Suicidal intent (XaA1G); Self-injurious behaviour (X766K); [X]Intent self harm by crash motor vehic occ unspecif place (U2Dz.); [X]Intentional self-harm (XE22c); Parasuicide (Xa04L); [X]Intentionl self harm by oth specif means occurrn at home (U2y0.); Attempted suicide - hanging (Xa3jC); [X]Self mutilation (U2E..); [X]Intent self harm by hanging strangulation / suffocation (U21..); Scratches self (X766T); Suicide and selfinflicted injury NOS (TKz..); Cutting own wrists (XaBAO) |
|  | Victim of abuse, bereavement or grief | Grief reaction (XE1Yo); Family bereavement (13M..); Victim of sexual abuse (Ua2AA); Bereavement (Ua1q5); Victim of domestic violence (XaLVA); Child abuse (SN55.); Victim of emotional abuse (XaLV9); History of domestic violence (XaJhe); Victim of domestic abuse (XaaSO); History of domestic abuse (XaN21); Family bereavement NOS (XE0pk); Other childhood and adolescent emotional problems (E2Dy.); History of abuse (XaEFq); Victim of abuse (Ua2A5); Victim of child sexual abuse (Ya0lb); History of sexual abuse (XaEFs); History of being victim of domestic violence (XaZhL); Victim of physical abuse (Ua2A7); History of emotional abuse (XaEFt); Unexpected bereavement (Ua1q1); Abnormal grief reaction (Ua18k); Sudden bereavement (Ua1q4); History of physical abuse (XaEFr); Victim of bullying (Ua2A3); Victim of verbal abuse (Ua2A9); [V]Personal history of neglect (XaPp6); Victim of other person's behaviour (Ua2A1); Feeling grief (Ua15r); Victim of child abuse (Ua2AE); Grief reaction ( & [bereavement reaction]) (E2900); Childhood emotional disorder (XE1Yv); Oppositional defiant disorder (E2Dy0); Victim of sexual activity without consent (XaXyb); History of domestic sexual abuse (Xaeyh) |
|  | Family history of mental health issues | History of postnatal depression (XaNg1); Child attention deficit disorder (E2E0.); Family history of mental disorder (128..); FH: Schizophrenia (1284.); Childhood schizophrenia NOS (X00S7); Behavioural and emotional disorder with onset in childhood (X00TR); Childhood emotional disorder (XE1Yv); Transient childhood tic (E2721); Childhood reactive attachment disorder (Eu941); Family history of autism (XaJid); FH: Manic-depressive state (1287.); FH: Mental disorder NOS (128Z.) |
| **Depression screening** | |  |
|  | Depression screening | Depression screening using questions (XaLIc); Depression monitoring invitation (Y1f1e); HAD scale: depression score (XaIwf); EuroQol five dimension five level anxiety depression score (XaYwr); Hospital anxiety and depression scale (XM0eK); Assessment using Whooley depression screen (XaY4X); Depression screening (6891.); Edinburgh postnatal depression scale declined (XaIKH); Depression anxiety stress scales depression score (XaKaX); Depression anxiety scale (XM0cP) |
|  | Mental health assessment | Mental health assessment (XaIYN); Perinatal mental health assessment (Y1e1c); Psychological assessment (Xabjw); Care Programme Approach assessment (Xa4HU); Emergency mental health assessment (XaIOg); Psychological wellbeing assessment (Xabr9) |
|  | Depression screening refused | Hospital Anxiety and Depression Scale declined (XadFQ); Edinburgh postnatal depression scale declined (XaIKH); Depression screening declined (XaboQ); Mental health assessment declined (XaXiE) |
| **Depression diagnosis** | |  |
|  | Depression diagnosis | Mixed anxiety and depressive disorder (X00Sb); Depressive disorder NEC (E2B..); Depression NOS (XaB9J); Depressive disorder (X00SO); H/O: depression (1465.); Single major depressive episode (XE1Y0); Reactive depression (XE1YC); Moderate depression (XaCIt); [X](Depressn: [episode unsp][NOS (& react)][depress dis NOS] (Eu32z); Postnatal depressive disorder (62T1.); Endogenous depression first episode (X00SS); [X]Moderate depressive episode (Eu321); On depression register (XaJWh); [X]Mild depressive episode (Eu320); [X]Depressive episode, unspecified (XE1Zb); [X]Recurrent depressive disorder (XE1Zc); [X]Other depressive episodes (XE1Za); Endogenous depression (X00SR); Mild depression (XaCIs); Severe postnatal depression (X40Dm); Severe depression (XaCIu); Agitated depression (X00SQ); Mild postnatal depression (X40Dl); Chronic depression (E2B1.); Moderate major depression (XSGol); [X]Mixed anxiety and depress disord (& mild anxiet depressn) (Eu412); [X]Severe depressive episode without psychotic symptoms (XE1ZY); [X]Recurrent depressive disorder, current episode moderate (Eu331); [X]Recurrent depressive disorder, current episode mild (Eu330); [X] Depression recurrent: [unspecified] or [monopolar NOS] (Eu33z); [X]Recurrent depressive disorder, unspecified (XE1Zf); Recurrent brief depressive disorder (Xa0wV); Severe major depression with psychotic features (XSGon); Major depressive disorder (XSEGJ); [X]Sev depress epis, no psych: (& single [agit][maj][vital]) (Eu322); Endogenous depression - recurrent (XM1GC); [X]Severe depressive episode with psychotic symptoms (XE1ZZ); Recurrent major depressive episodes (XE1Y1) |
| **Psychological therapies** | |  |
|  | Referral or seen by a psychologist or a counsellor, counselling, psychosocial support or therapies | Referral for mental health counselling (XaAen); Referral for guided self-help for depression (XaL0r); Referral to improving access to psychological therapies prog (XaPvw); Referral to psychology service (XaBvV); Referral to mental health counsellor (XaAfJ); Referral to mental health counselling service (XaAem); Cancer emotional and psychosocial support and advice (XaKI3); Cognitive - behaviour therapy (XaABO); Emotional and psychosocial support and advice (XaIpc); Psychological counselling (6779.); Digital cognitive behavioural therapy for depression (Y3f53); Psychotherapy (X71bp); Psychological therapies (XaIOt); Cognitive behavioural therapy NOS (XaM2L); Postnatal depression counselling (XaIpB); Problem solving therapy (XaItk); Attended psycho-educational group (XaKc7); Generic cognitive behavioural therapy (Xa8I9); Guided self help cognitive behavioural therapy (XaQC0); Psychosexual counselling (677A.); Cognitive analytic therapy (Xa8IX); Computerised cognitive behavioural therapy (XaKzQ); Other psychotherapy NOS (8G9Z.); IAPT (Improvin Access Psych Therapies) low intensity therapy (XaeHl); General psychotherapy (8G1..); Cognitive and behavioural therapy (Ub0qp); IAPT (Improvin Acces Psych Therapies) high intensity therapy (XaeHm); Other specified cognitive behavioural therapy (XaM2K); Positive feedback approach (XaDAc); Provocative therapy (8G41.); Psychosexual therapy (Xa8Il); Behavioural activation therapy (XaQ7n) |
|  | Referral or seen by a psychiatry or other mental health team | Referral to community mental health team (XaIkd); Referral to psychiatrist (XaBTX); Seen in psychiatry clinic (9N1T.); Referral to older age community mental health team (XaL0p); Referral to psychiatry service (8H49.); Private referral to psychiatrist (8HVO.); Psychiatric self-referral (8HJ3.); Referral by community mental health nurse (XaJQZ); Referral to rehabilitation psychiatrist (XaAgF) ; Seen in psychiatry clinic (9N1T.); Seen in mental health clinic (XaONq); Seen by primary care graduate mental health worker (XaL0t); Seen by community mental health nurse (XaAUA); Seen by psychiatrist (XaATC); Seen by mental health triage nurse (XaL0u); Seen by primary care mental health team (XaZrh); Seen by consultant psychiatrist (XaZsy); Seen by liaison psychiatrist (XaATF); Seen by primary care mental health gateway worker (XaM7s) |
|  | Referral refused or missed appointment | Did not attend mental health appointment (XaIuR); DNA - Did not attend mental health review (XaLIb); Referral to IAPT (imp acc to psych thera) programme declined (XaZUd); Did not attend psychiatry clinic (XaJpO); Did not attend depression review (XaaZg); Did not attend psychotherapy appointment (XaXHm); Did not attend cognitive behaviour therapy (XaLCQ); Did not attend community psychiatric nurse review (XaK1f); Did not attend counselling appointment (XaaWG) |
| **Social prescribing** | |  |
|  | lifestyle advice, social prescribing offer and referral to social and sport activities | Health education - exercise (6798.); Advice about exercise (Xa9zF); Lifestyle advice regarding exercise (XaJIt); Signposting to social prescribing service (XagOR); Advice about psychological well-being (XaIyv); Social prescribing offered (XaaEA); Social prescribing declined (XaaEB); Patient given advice about management of depression (XaKEz); Social prescribing for mental health (XaQvz); Referral to social prescribing service (XaaEC); Referral to physical activity programme (XaIQY); Referral for physical activity service offered (XaXR3); Participation in Tai Chi (Ub1Jo) |
|  | Social prescribing declined | Social prescribing declined (XaaEB) |
| **Depression outcomes** | |  |
|  | normal/stable mood or controlled depression | Normal mood (XaIJ4); Mood stable (Ua1r8); Feeling calm (Ua16A); Cheerful mood (Ua1X9); Feeling relief (Ua169); Feeling content (XM018); Feeling excited (Ua15w); Positive thoughts (Ua1rI) |
|  | depression remission/resolved | Patient in early remission (XaKU2); Depression resolved (XaLG0); Patient in remission (XaEKK); Patient in full remission (XaKU4); [X]Recurrent depressive disorder, currently in remission (Eu334); Patient in partial remission (XaKU3) |
